# Unexpected coccidioidomycosis presenting as lung nodules with presumptive diagnosis of malignancy

**DOI:** 10.1002/ccr3.8316

**Published:** 2023-12-09

**Authors:** Jennifer Cai, David Sin, Sarah Tomassetti

**Affiliations:** ^1^ Department of Pathology Harbor‐UCLA Medical Center Torrance California USA; ^2^ University of California at Irvine Irvine California USA; ^3^ Department of Radiology Harbor‐UCLA Medical Center Torrance California USA; ^4^ Division of Hematology and Oncology Harbor‐UCLA Medical Center Torrance California USA; ^5^ David Geffen School of Medicine at UCLA Los Angeles California USA

**Keywords:** coccidioides, coccidiomycosis, lung nodule, malignancy, pulmonary nodule

## Abstract

Coccidioidomycosis can present as fluorodeoxyglucose (FDG) avid lung nodules which may be mistaken as relapse in patients with a history of malignancy. Detailed clinical history, relevant laboratory testing, and/or tissue biopsy with histologic evaluation are necessary for correct diagnosis.

## Graphical abstract

Unexpected coccidioidomycosis presenting as lung nodules in a patient with a history of malignancy.

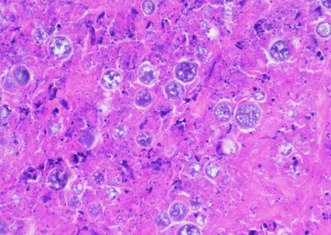

## 1 INTRODUCTION

According to the Fleischner Society glossary of terms for chest imaging, a lung nodule is an approximately rounded opacity, which may be well‐ or less well‐defined, measuring up to 3 cm in diameter.^1^ Lung nodules can be either benign or malignant. Rounded pulmonary lesions larger than 3 cm in diameter are termed lung masses, which are more likely to represent lung cancer.^2^ The lung is one of the most common sites of malignant metastasis. Multiple lung nodules in patients with nonpulmonary malignancies are highly suspicious for metastasis.^3^ Here, we describe the case of a 60‐year‐old female with a history of hepatocellular carcinoma and diffuse large B‐cell lymphoma, in whom radiographic imaging surveillance showed pulmonary nodules concerning for malignancy, and the subsequent histologic diagnosis of coccidioidomycosis was unexpected.

## 2 CASE

**FIGURE 1:**
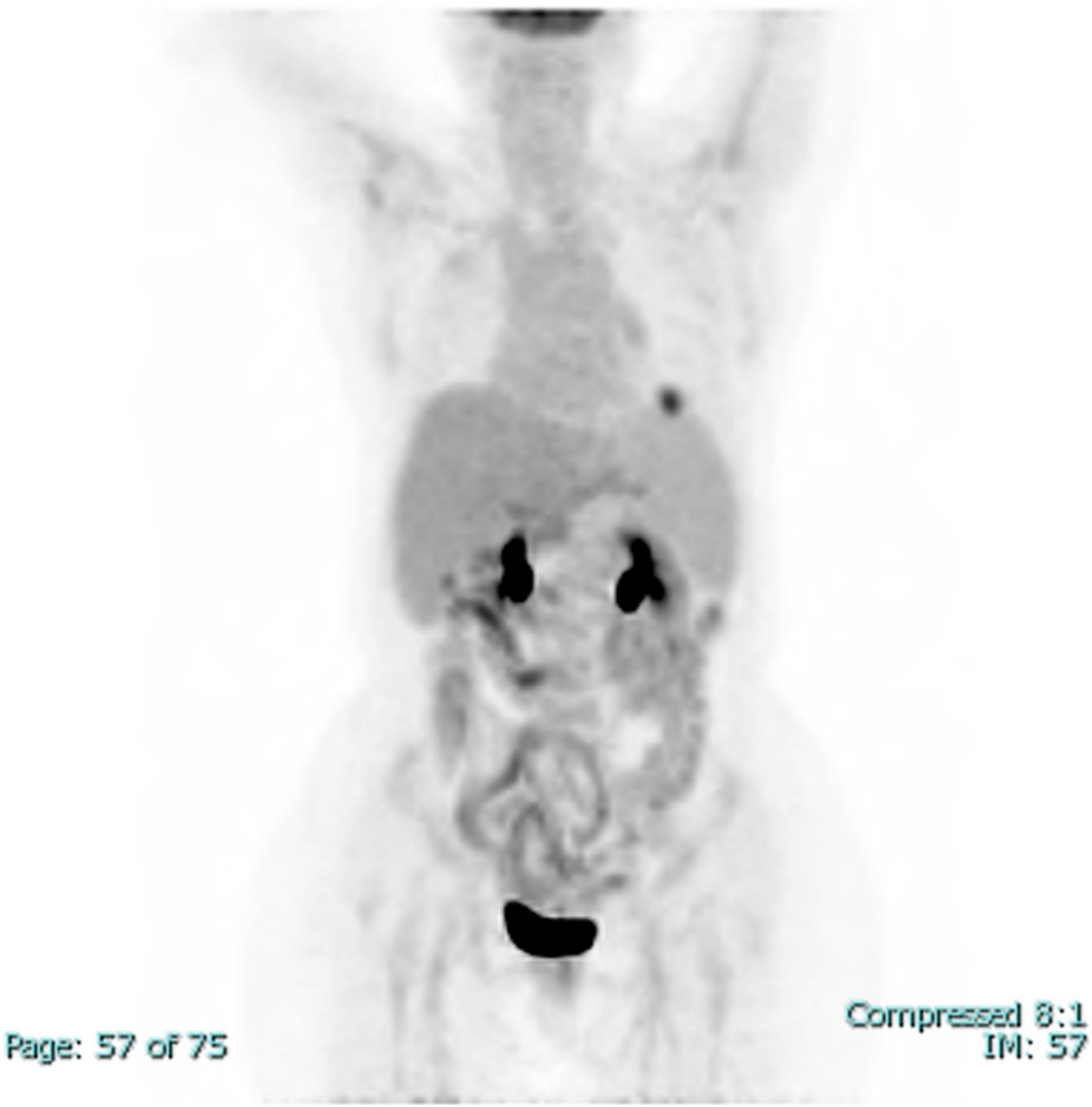
A coronal maximal intensity projection (MIP) fluorodeoxyglucose positron emission tomography (FDG PET) shows a 2.5 cm FDG avid pulmonary nodule in the left lower lobe.

**FIGURE 2:**
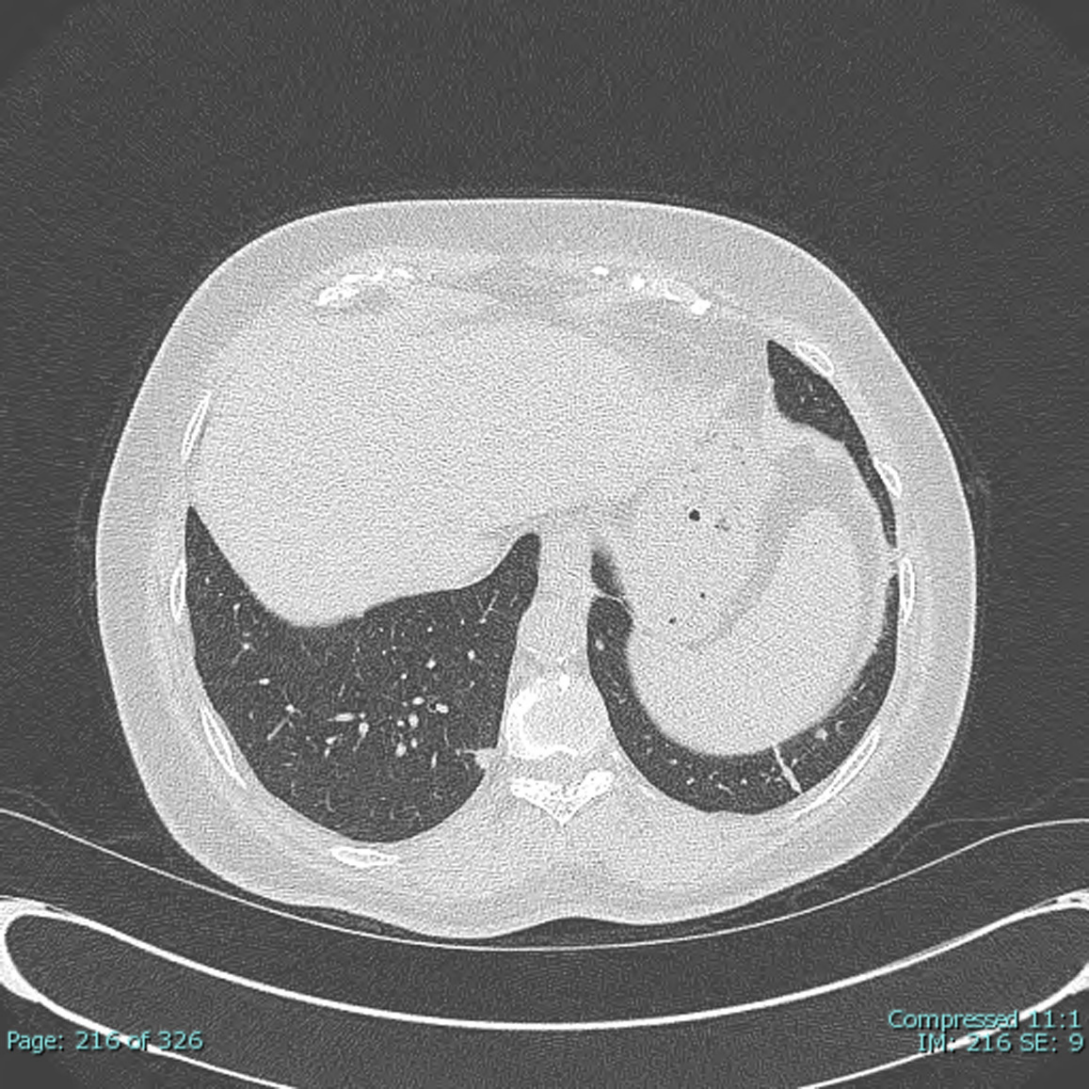
An axial noncontrast computed tomography (CT) slice at the lower chest in the lung window shows a new 6 mm juxta pleural nodule along the medial margin of the right lower lobe.

**FIGURE 3:**
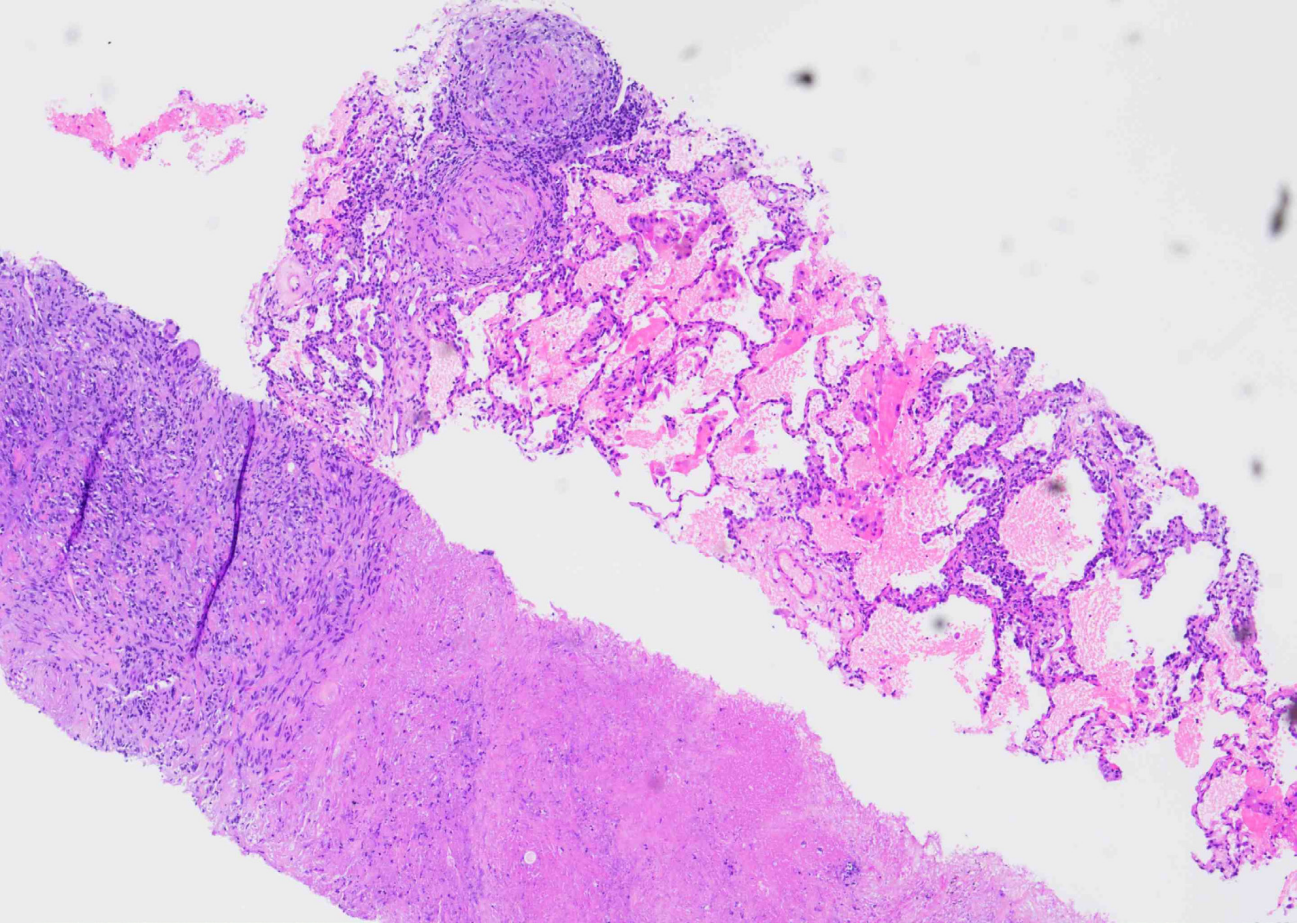
Photomicrograph of the computed tomography (CT)‐guided core biopsy of the lung nodule. The lung tissue is largely replaced by granulomas with or without central necrosis. (H&E stain, original magnification, × 40).

**FIGURE 4:**
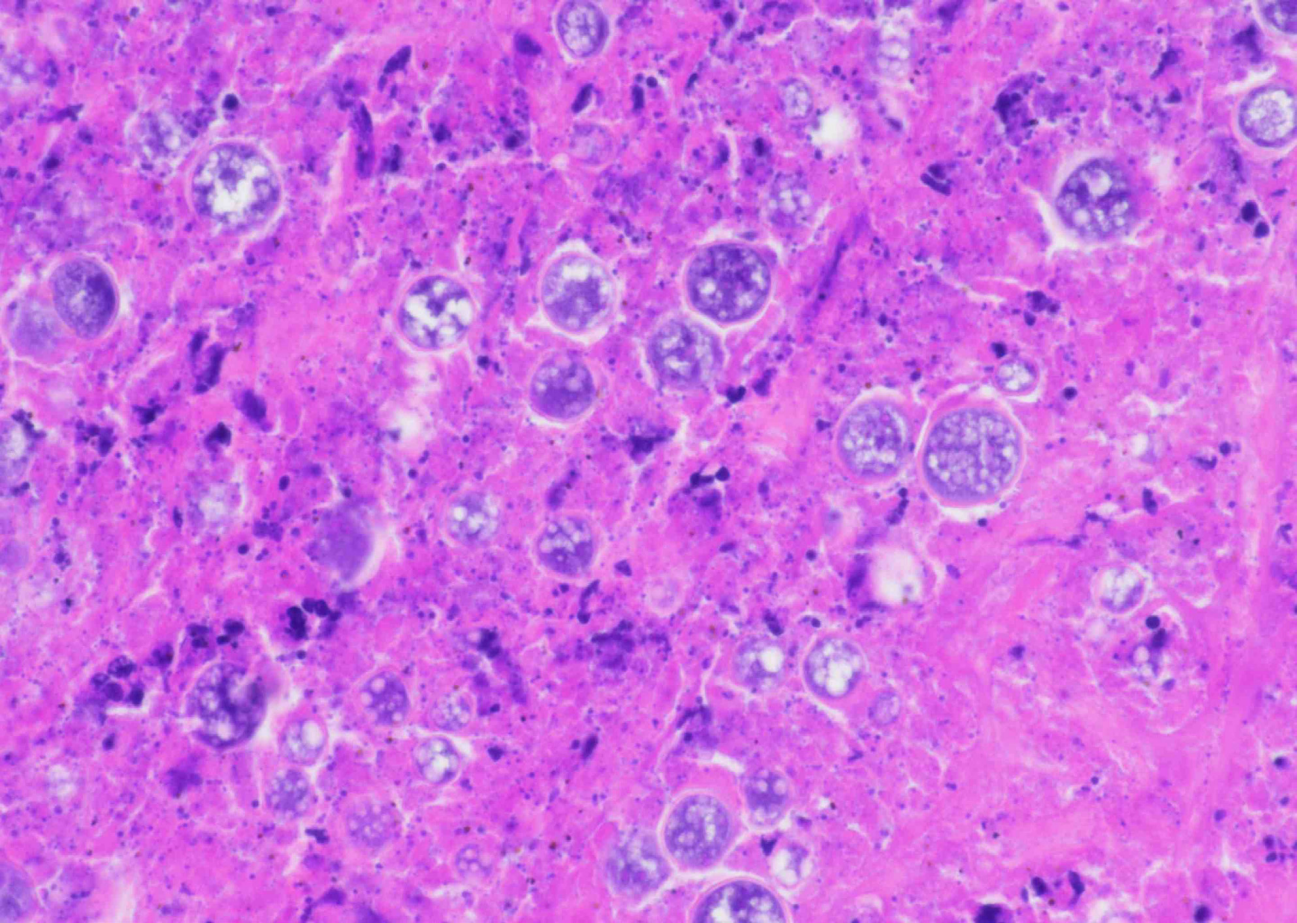
Photomicrograph of the computed tomography (CT)‐guided core biopsy of the lung nodule. There are numerous *Coccidioides* spherules containing endospores in the necrotic centers of the granulomas (H&E stain, original magnification, × 400).

**FIGURE 5:**
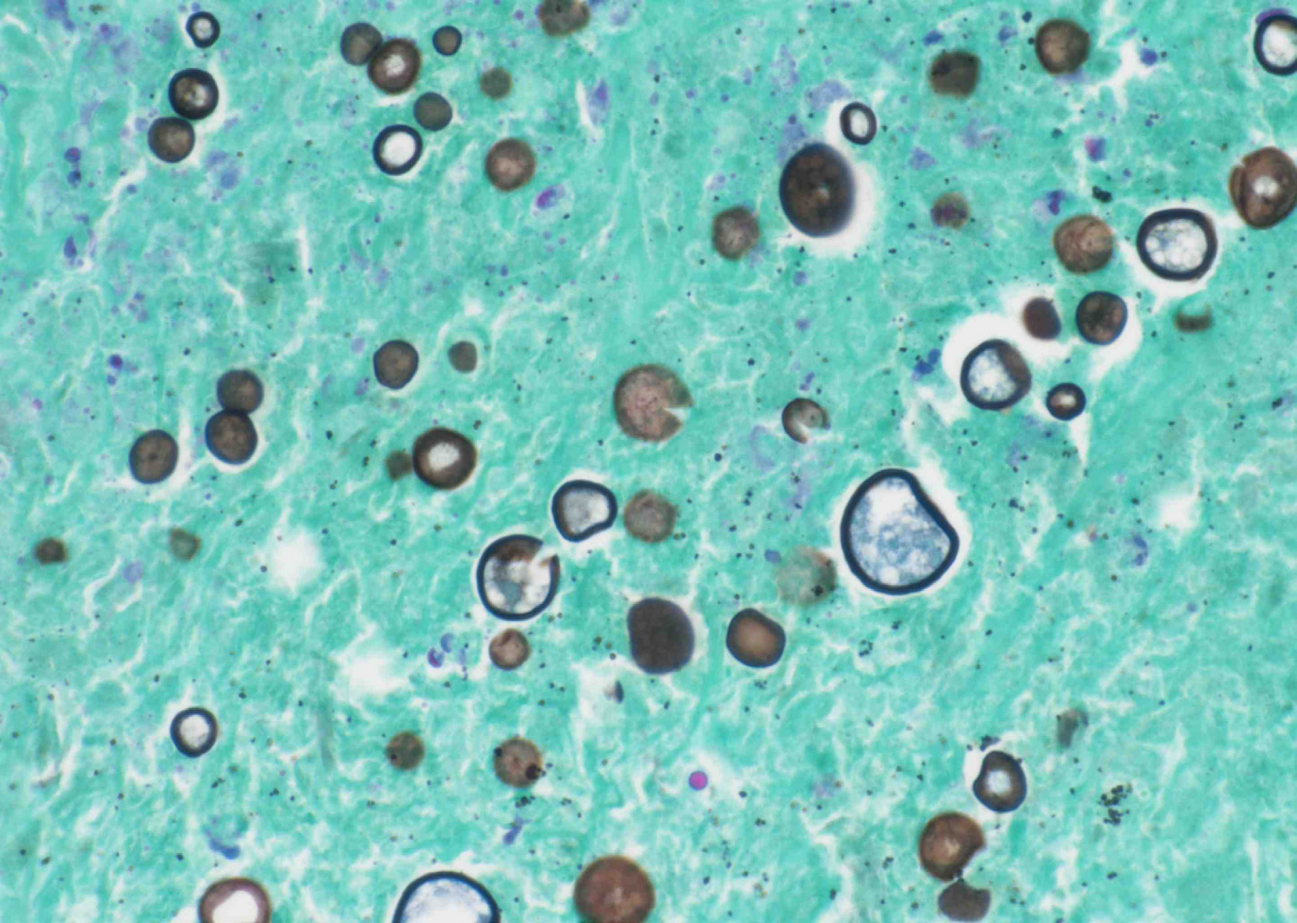
Photomicrograph of the computed tomography (CT)‐guided core biopsy of the lung nodule. The Grocott–Gömöri's methenamine silver stain highlights the *Coccidioides* spherules (original magnification, × 400).

**FIGURE 6:**
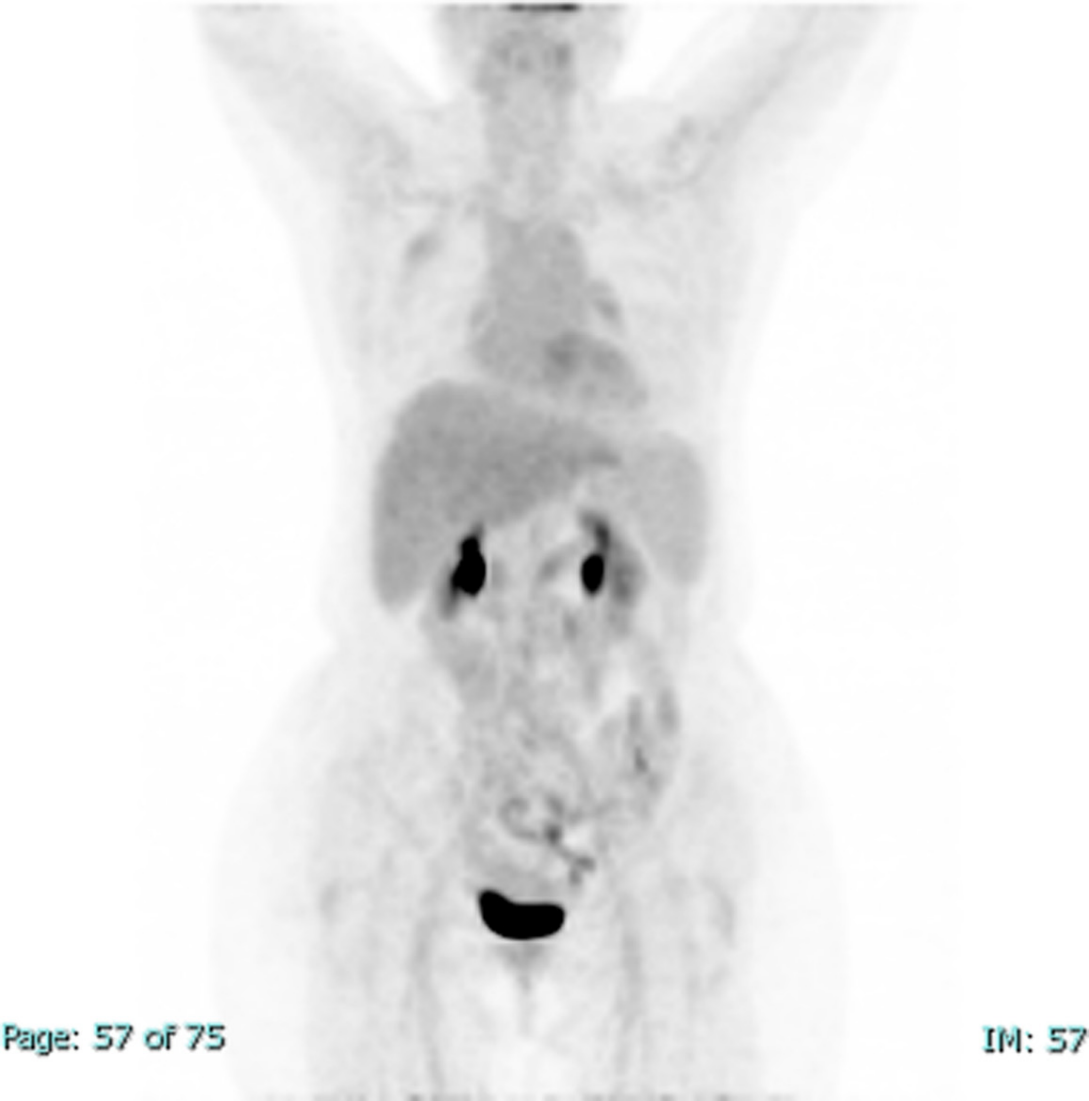
A coronal maximal intensity projection (MIP) fluorodeoxyglucose positron emission tomography (FDG PET) following fluconazole therapy shows that the FDG avidity of the pulmonary nodule in the left lower lobe significantly decreased to background physiologic levels.

A 60‐year‐old HIV‐negative woman with a medical history of hepatitis C cirrhosis, diffuse large B‐cell lymphoma, hepatocellular carcinoma, diabetes mellitus, hypothyroidism, chronic kidney disease, and depression presented to oncology clinics for follow‐up. She denied night sweats, fever, chills, headache, chest pain, palpitations, shortness of breath, abdominal pain, nausea, vomiting, and any recent weight loss. She was diagnosed with diffuse large B‐cell lymphoma 10 years earlier and achieved complete remission after 6 cycles of chemotherapy with R‐CHOP (Rituximab, Cyclophosphamide, Hydroxydaunorubicin, Oncovin, and Prednisone). Her lymphoma relapsed in gingiva 2 years after the initial diagnosis and was treated with 4 cycles of chemotherapy with RICE (Rituximab, Ifosfamide, Carboplatin, and Etoposide phosphate). Four years later, she was found to have a liver lesion which was considered clinically and radiographically consistent with hepatocellular carcinoma based on the hepatic multiphase computed tomography (CT) findings and elevated serum alpha‐fetoprotein (AFP) level in the setting of cirrhosis. She was treated with transarterial chemoembolization (TACE). After 1 year, she developed a second relapse of her lymphoma in the liver and was treated with 13 cycles of R‐GemOx (rituximab, gemcitabine, and oxaliplatin) with a complete metabolic response. At the current presentation, to monitor her malignant diseases, a surveillance whole‐body Positron emission tomography‐computed tomography (PET/CT) scan was performed and showed a new, fluorodeoxyglucose (FDG) avid left lower lobe pulmonary nodule measuring 2.5 cm in diameter with an SUV max of 8.6 (Figure 1), concerning for malignancy. Her complete blood count (CBC) with automatic differential 3 days before the PET/CT showed mild normocytic anemia (Hgb 11.1 g/dL, MCV 87.5 fL), mild leukopenia (WBC 4.2 K/cumm, with 75.3% neutrophils, 15.5% lymphocytes, 7.9% monocytes, 1.0% eosinophils, and 0.3% basophils), and moderate thrombocytopenia (56 K/cumm). A follow‐up CT of her thorax 2 months after the PET‐CT demonstrated that the lung nodule had decreased in diameter to 1.5 cm, but with multiple additional new bilateral lung micronodules ranging from 1 to 6 mm in diameter (Figure 2). Although the decrease in nodule size raised the possibility of infection, given the multiple new bilateral pulmonary micronodules and the high clinical suspicion for malignancy, a CT‐guided core biopsy of the largest lung nodule was performed, which showed both necrotizing and non‐necrotizing granulomas (Figure 3). Many *Coccidioides* spherules were seen in the necrotic centers of the granulomas, ranging in size from 20 to 200 microns in diameter, and containing endospores (Figure 4). The Grocott–Gömöri's methenamine silver (GMS) stain highlighted *Coccidioides* spherules (Figure 5). The acid‐fast bacteria (AFB) stain was negative for acid‐fast bacilli. By immunohistochemistry, many CD3‐positive small T‐cells were seen, with virtually no B‐cells by CD20 and PAX‐5 stains. The findings were consistent with coccidioidomycosis. There was no evidence of lymphoma or carcinoma. Her serum *Coccidioides* Serology Panel (Quest Diagnostics Nichols Institute, San Juan Capistrano, CA) showed an antibody titer of 1:2 (Complement Fixation, reference range: < 1:2), but with negative antibodies to Coccidioides F antigen (IgG Immunodiffusion) and TP antigens (IgM Immunodiffusion). Antibodies to *Coccidioides* were not detected in her cerebrospinal fluid (CSF) by either complement fixation or immunodiffusion. She also denied joint swelling or pain. The patient underwent 8 months of fluconazole treatment. A follow‐up PET/CT scan after 6 months of treatment showed decreased FDG uptake of the left lower lobe pulmonary nodule (no change in size, still 2.5 cm in diameter) with an SUV max of 2.2, and no evidence of hypermetabolic disease recurrence (Figure 6). As of 10 months postcompletion of antifungal therapy, her bilateral lung lesions remain stable by serial CT scans and magnetic resonance imaging (MRI), without signs of recurrence of malignancy. The size of the left lower lobe pulmonary nodule remained at 2.2–2.3 cm in diameter by CT and at 2.4 cm in diameter by MRI.

## 3 DISCUSSION

An FDG PET scan is commonly used to detect metabolically active malignant lesions and may be used to more accurately stage malignant diseases and to monitor the therapy response of malignant diseases. However, although it is especially helpful in detecting metastatic malignancy, FDG‐positive lesions can also be seen in nonmalignant conditions including infections such as coccidioidomycosis,^4^ inflammations, autoimmune disorders, sarcoidosis, and benign tumors.^5^ If these conditions are not identified clinically and/or by tissue biopsies, misdiagnosis can lead to inappropriate therapies. Our case describes an FDG‐positive lung nodule in a patient with a history of both diffuse large B‐cell lymphoma and hepatocellular carcinoma. Given the malignant history and the lack of symptoms and laboratory evidence of lung infection, relapsed malignancy was highly suspected. This case emphasizes the importance of tissue biopsy and histologic evaluation in patients diagnosed with FDG avid lesions.

Coccidioidomycosis, also known as cocci or Valley fever, is a disease caused by the dimorphic fungus *Coccidioides immitis* or *Coccidioides posadasii* which exists either as mycelia in the soil or as spherules in the lung and other tissues. Infections are established mainly through inhalation of aerosolized arthrospores into the lung or less likely through direct skin contact^6^ or transplanted organs.^7^ Hematogenous extrapulmonary systemic dissemination occurs in 1% of infections.^8^ Coccidioidomycosis is endemic to the southwestern United States, northern Mexico, and South America.^6^, ^8^ Upon further inquiry after the histologic diagnosis was made, our patient admitted that she had traveled to Arizona a few months before the finding of her pulmonary nodule. The clinical manifestations of coccidioidomycosis vary. Symptoms may include fever, cough, shortness of breath, chest pain, headache, weight loss, skin rash, and migratory arthralgias.^9^ However, most infections are asymptomatic, as seen in the present case. In disseminated coccidioidomycosis, central nervous system (CNS) involvement is often seen, which can be fatal. Thus, it is important to rule out CNS involvement by CSF study.

Both asymptomatic and symptomatic pulmonary coccidioidomycosis can result in radiographically visible lung nodules^7^ and it is difficult to differentiate lung nodules due to coccidioidomycosis from those due to malignancy radiographically. Cavitary nodules, satellite nodules, and chronic lung disease, in patients from endemic regions may support coccidioidomycosis rather than primary lung cancer.^10^ Definitive diagnosis of coccidioidomycosis relies on careful clinical evaluation and laboratory diagnostic testing. Young age (< 55 years old), absence of past lung diseases, a job in the farming or construction sector, and/or traveling in the endemic regions will raise the possibility of coccidioidomycosis.^11^ Microbiological culture, histopathological evaluation, or serological testing should be used to confirm the diagnosis.^12^ Serological assays such as enzyme immunoassay (EIA), immunodiffusion, and complement fixation may be less reliable^3^, ^13^, ^14^ and often a biopsy is required to establish the diagnoses via histopathology, culture, and possibly PCR. Histologically, granulomas are seen in all cases, with or without necrosis.^15^ The presence of endospore‐containing spherules is diagnostic of coccidioidomycosis. In our patient, the biopsy of her pulmonary nodule showed many necrotizing and non‐necrotizing granulomas; numerous spherules containing endospores were seen in the necrotic centers of the granulomas, supporting the diagnosis of coccidioidomycosis.

It is challenging to differentiate between *Coccidioides immitis* and *Coccidioides posadasii* because they have identical morphology and similar phenotypes. Fortunately, this differentiation is unnecessary as the two species seem to have almost identical clinical presentations and antifungal susceptibility profiles.^16^

Asymptomatic pulmonary nodules attributed to coccidioidomycosis in an immunocompetent patient do not require treatment.^14^ However, azole antifungals may be used in other patients depending on patient risk factors, serologic studies, and characteristics of the lesions.^14^ Intravenous amphotericin B should be reserved only for those with severe disease.^14^, ^17^ Current therapies do not eradicate *Coccidioides* species from the lesions of chronic coccidioidal pneumonia and symptoms may recur.^14^ Thus, regular follow‐up is an important component in the management of coccidioidomycosis, to confirm that the infection remains uncomplicated.^14^ As long as repeated radiographic imaging demonstrates the lesions are stable over time and the disease shows a benign clinical course, further intervention is unnecessary. Whether or not antifungal therapy is administered, the infection will eventually resolve in uncomplicated cases.

In conclusion, although lung nodules, especially FDG avid ones, in patients with a history of malignancy warrant a high suspicion for malignant recurrence, clinicians should still maintain vigilance for excluding other potentially treatable infectious etiologies, even in patients without symptoms of infections because infections such as coccidioidomycosis tend to manifest without symptoms. Detailed clinical history, relevant laboratory testing, and/or tissue biopsy with histologic evaluation are necessary for accurate diagnosis.

## AUTHOR CONTRIBUTIONS

**Jennifer Cai:** Conceptualization; investigation; methodology; project administration; visualization; writing – original draft; writing – review and editing. **David Sin:** Methodology; resources; visualization; writing – review and editing. **Sarah Tomassetti:** Investigation; project administration; resources; writing – review and editing.

## FUNDING INFORMATION

None.

## ETHICS STATEMENT

Ethical review and approval of the study are not applicable in this case.

## CONSENT

Written informed consent was obtained from the patient to publish this report in accordance with the journal's patient consent policy.

## Data Availability

No datasets were generated or analyzed during the current study.

## References

[1] Hansell DM , Bankier AA , MacMahon H , McLoud TC , Müller NL , Remy J . Fleischner society: glossary of terms for thoracic imaging. Radiology. 2008;246(3):697‐722. doi:10.1148/radiol.2462070712 18195376

[2] Loverdos K , Fotiadis A , Kontogianni C , Iliopoulou M , Gaga M . Lung nodules: a comprehensive review on current approach and management. Ann Thorac Med. 2019;14(4):226‐238. doi:10.4103/atm.ATM_110_19 31620206 PMC6784443

[3] Jamil A , Kasi A . Lung Metastasis. StatPearls [Internet]. StatPearls Publishing; 2023 Available from: https://www.ncbi.nlm.nih.gov/books/NBK553111/ 31971751

[4] Dryden JR , Starsiak MD , Johnston MJ , Silverman ED . Bone scan, PET‐CT, and MRI in disseminated coccidioidomycosis. Clin Nucl Med. 2017;42(4):319‐322. doi:10.1097/RLU.0000000000001570 28166146

[5] Safaie E , Matthews R , Bergamaschi R . PET scan findings can be false positive. Tech Coloproctol. 2015;19:329‐330. doi:10.1007/s10151-015-1308-3 25939996

[6] Ahmad F , Patel K , De Leon JC , Buttacavoli FA . Disseminated coccidioidomycosis of the knee joint requiring synovectomy and arthrotomy. J Orthop Case Rep. 2021;11(2):76‐80. doi:10.13107/jocr.2021.v11.i02.3034 PMC8180333 34141676

[7] Johnson L , Gaab EM , Sanchez J , et al. Valley fever: danger lurking in a dust cloud. Microbes Infect. 2014;16:591‐600. 25038397 10.1016/j.micinf.2014.06.011 PMC4250047

[8] Deus Filho A . Chapter 2: coccidioidomycosis. J Bras Pneumol. 2009;35(9):920‐930. doi:10.1590/s1806-37132009000900014 19820819

[9] Akram SM , Koirala J . Coccidioidomycosis. StatPearls Publishing; 2023 Available from: https://www.ncbi.nlm.nih.gov/books/NBK448161/ 28846274

[10] Peterson MW , Jain R , Hildebrandt K , Carson WK , Fayed MA . Differentiating lung nodules due to Coccidioides from those due to lung cancer based on radiographic appearance. J Fungi (Basel). 2023;9(6):641. doi:10.3390/jof9060641 37367577 PMC10302563

[11] Alčauskas T , Zablockienė B , Zablockis R , Svetikas L , Bilotaitė L , Jančorienė L . Pulmonary coccidioidomycosis: a case report and literature review. Medicina (Kaunas). 2022;58(5):655. doi:10.3390/medicina58050655 35630071 PMC9143117

[12] Thompson GR , Le T , Chindamporn A , et al. Global guideline for the diagnosis and management of the endemic mycoses: an initiative of the European Confederation of Medical Mycology in cooperation with the International Society for Human and Animal Mycology. Lancet Infect Dis. 2021;21(12):e364‐e374. doi:10.1016/S1473-3099(21)00191-2 Epub 2021 Aug 6. Erratum in: Lancet Infect Dis. 2021 Nov;21(11):e341. PMID: 34364529; PMCID: PMC9450022. 34364529 PMC9450022

[13] Blair JE , Coakley B , Santelli AC , Hentz JG , Wengenack NL . Serologic testing for symptomatic coccidioidomycosis in immunocompetent and immunosuppressed hosts. Mycopathologia. 2006;162(5):317‐324. doi:10.1007/s11046-006-0062-5 17123029 PMC2780641

[14] Galgiani JN , Ampel NM , Blair JE , et al. 2016 Infectious Diseases Society of America (IDSA) clinical practice guideline for the treatment of coccidioidomycosis. Clin Infect Dis. 2016;63(6):e112‐e146. doi:10.1093/cid/ciw360 27470238

[15] Ricciotti RW , Shekhel TA , Blair JE , Colby TV , Sobonya RE , Larsen BT . Surgical pathology of skeletal coccidioidomycosis: a clinical and histopathologic analysis of 25 cases. Am J Surg Pathol. 2014;38(12):1672‐1680. doi:10.1097/PAS.0000000000000284 25007149

[16] Saubolle MA , McKellar PP , Sussland D . Epidemiologic, clinical, and diagnostic aspects of coccidioidomycosis. J Clin Microbiol. 2007;45(1):26‐30. doi:10.1128/JCM.02230-06 17108067 PMC1828958

[17] Ampel NM . The treatment of coccidioidomycosis. Rev Inst Med Trop Sao Paulo. 2015;57 Suppl 19(Suppl 19):51‐56. doi:10.1590/S0036-46652015000700010 26465370 PMC4711193

